# The Gold Coast Integrated Care Model

**DOI:** 10.5334/ijic.2233

**Published:** 2016-07-08

**Authors:** Martin Connor, Helen Cooper, Anne McMurray

**Affiliations:** 1 Professor, Menzies Health Institute, Executive Director Centre for Health Innovation, Griffith University and Gold Coast Hospital and Health Services, 1 Hospital Blvd, Southport, Queensland, 4215, Australia; 2 BN, GDipAcuteCareN, MBA, Managing Director Gold Coast Integrated Care, Gold Coast Hospital and Health Service, Level 1, 8 High Street, Southport, Qld, 4215, Australia; 3 Professor, Menzies Health Institute, Principal Research Fellow, Centre for Health Innovation, Griffith University, Level 1, 8 High Street, Southport, Qld, 4215, Australia

**Keywords:** integrated care, primary care, acute care, health reform, Australia

## Abstract

This article outlines the development of the Australian Gold Coast Integrated Care Model based on the elements identified in contemporary research literature as essential for successful integration of care between primary care, and acute hospital services. The objectives of the model are to proactively manage high risk patients with complex and chronic conditions in collaboration with General Practitioners to ultimately reduce presentations to the health service emergency department, improve the capacity of specialist outpatients, and decrease planned and unplanned admission rates. Central to the model is a shared care record which is maintained and accessed by staff in the Coordination Centre. We provide a process map outlining the care protocols from initial assessment to care of the patient presenting for emergency care. The model is being evaluated over a pilot three year proof of concept phase to determine economic and process perspectives. If found to be cost-effective, acceptable to patients and professionals and as good as or better than usual care in terms of outcomes, the strategic intent is to scale the programme beyond the local health service.

## Introduction

The urgent and critical mandate for health reform is clearly articulated throughout the Australian and global communities [1,2,3,4,5]. Over the past decade a number of state and Commonwealth reviews, including the Forster Review of the Queensland Health System, have identified that health services are inequitable, costly, poorly integrated and unsustainable, particularly with population ageing and the exponential growth of chronic diseases [3,6,7,8,9,10,11]. The Australian National Health and Hospitals Reform Committee strongly recommended three immediate and crucial responses to redress these issues: a focus on access and equity, vertical and horizontal service integration, and development of an agile, self-improving and sustainable health system focused on primary health care [3]. These systemic improvements can be met within an appropriate and adaptable model of integrated care, defined by the WHO [5] as the organisation and management of health services so that people get the care they need, when they need it, in ways that are user friendly, achieve the desired results and provide value for money. Integrated care is central to the National Chronic Disease Strategy and the Queensland Strategy for Chronic Disease, both of which have allocated substantial investments to meet the needs of those with chronic and complex conditions [12,13]. Patients with chronic conditions have considerable unmet needs as they typically have to access sequential or simultaneous services from multiple providers in different locations or with culturally appropriate care provisions. They may also be subject to situations where clinicians and services lack the capacity to work effectively together; or where there is a lack of structures or clinical governance systems to support integration, such as unreliable referral systems, inconsistent eligibility criteria, no electronic records or secure information sharing [14]. In response to these barriers to service delivery we describe the development of an integrated care model characterised by a collaborative, holistic, and patient-centred approach to meeting the health needs of the population with chronic and complex conditions throughout the Gold Coast Hospital and Health Services (HHS) and the local community of multidisciplinary primary health care providers.

## Integrated Care Models

A review of American models such as Kaiser Permanente and Intermountain Health outlines the merits of integrated care programmes that focus on high-impact health conditions, such as those experienced by the vulnerable population with chronic and complex conditions [15,16,17,18,19]. These organisations use a primary care hub, such as a coordinating centre for care where a ‘transition coach’, or ‘care navigator’ can optimise the care path provided by members of the multidisciplinary team in collaboration with primary care practitioners. Evaluation of the American experience shows that a single disease focus is problematic, given the comorbidities of most people with chronic conditions [20]. Successful integrated care programmes are based on a number of features including models where patients are partners in care with self-management support, multidisciplinary care pathways organised through a single point-of-entry, comprehensive services, case management, an organised provider network, defined referral and service procedures, enhanced information management and pooling of funds [18]. The American Patient-Centred Medical Home is an example of this type of approach that clearly situates primary care at the centre of chronic illness management [11,21,22,23]. The medical home acts as a Coordination Centre for patients and their families to provide easy access to first-contact, comprehensive care where patients develop an ongoing relationship with the care team and participate fully in care planning [21]. Evaluations of the patient-centred medical home indicate that future developments need to focus on population health; that is, families and communities as well as individuals, which has been a missing element in some models of primary care [24]. This recommendation is more closely aligned with the global understanding of primary health care as distinct from primary care [5].

The patient-centred medical home concept has been considered in Australia by the Commonwealth Government and the Royal Australian College of General Practitioners. A systematic review indicates that the model is concordant with the College’s directions for the future; however its success would require substantial organisational change management from a physician-centred to a team approach, and transformation of payment systems as well as accreditation measures and targets [25]. Janamanian et al [25] suggest that the medical home model could be achieved through a partnership between College and the Australian Commission on Safety and Quality in Health Care, who are strong advocates for patient-centred care as a way of maintaining quality and safety [26]. The Australian Healthcare and Hospitals Association have begun a dialogue on policy initiatives that impact on health services and system integration, including consideration of providing bundled care packages for chronic diseases, having private insurance funding for primary care providers, and the implications of the transition from Medicare Locals to Primary Health Networks (PHNs) [27]. They concede that first, there is a need for sufficient evidence, data and funding to support bundled care packages and integration of services more broadly. Second, given the public/private divide in Australia’s health insurance system, any changes in private health insurance needs to avoid disadvantaging those without private health insurance. Third, there is potential for the PHN networks to play a major role in ensuring a dual focus on disease management and illness prevention if they are given the flexibility to organise and plan a well-integrated primary health sector [27]. In examining the feasibility of Australian programmes the Primary Healthcare Research and Information Service has suggested a focus on the patient journey and outcomes, particularly in helping people become health literate [28]. Data sharing is seen as a crucial factor in influencing the adoption of integrated care models in Australia, given historical inconsistencies in sharing electronic medical records [29,30,31]. The key to data sharing between acute and primary care settings lies in ensuring that medical home/Coordination Centre staff have access to patient data from all of these settings.

Researchers and policy-makers in the United Kingdom have a long standing interest in models of integrated care, which is somewhat easier in the context of decentralised, capitated health services than in the Australian fee-for-service model. Their research underlines the importance of strong leadership in programme development, and the centrality of the multidisciplinary team in implementing them [32,33,34,35,36]. Evaluations of studies in the United Kingdom demonstrate the importance of evidence-based planning that includes both economic and process evaluations. Data include agreed system-wide metrics for defining success and monitoring performance, including the distinct and mutual learning needs of the healthcare team, given differences in organisational and disciplinary backgrounds [37,38]. Evaluations also show the need for overarching governance arrangements, and adequate infrastructure, especially information and communications technology [39]. Petch et al [40] also recommend a nuanced approach to evaluation to yield more meaningful data than simply analysing cost effectiveness. They suggest the need to focus on outcomes, not targets; cultures, not just structures; place, not organisation; delegation, not transfer of functions; and an emphasis on clinical and professional engagement.

## The Gold Coast Integrated Care Model

The Gold Coast Integrated Care Model was developed over an 18 month period from September 2013 to March 2015 to improve services to the local population with chronic and complex conditions. During the design phase, consideration was given to various international models of integrated care, including the Trafford health model in which the first author played a significant role [34]. It was designed to operate on the macro (shared governance between care organisations), meso (disease status or sub-population types) and micro level (organising care around individual patient needs) as recommended by Curry and Ham [35] from their evaluation of successful integrated care programmes in the United Kingdom. This multi-level design is guided by Valentijn’s [41] conceptual framework linking all three levels through aligned functional and normative integration. In our case, the whole-system shared governance model provides an envelope within which patient-centred care is enabled by holistic assessment of individual risk stratification. Our approach to risk stratification is based on the effectiveness of Wagner’s (2001) chronic care model in guiding proactive systems of shared care for managing chronic diseases [15,16,42]. The objectives of the model are to proactively manage high risk patients in close collaboration with general practitioners

(GPs) aimed at reducing presentations to the health service emergency department, improve the capacity of specialist outpatients, and decrease admission rates (planned and unplanned). Our collaborative approach revolves around the centrality of the patient (and family) working closely with the primary practitioner (GP), and members of the multidisciplinary team (MDT), which includes nurses, pharmacists and other allied health professionals. The objective of the collaboration is to bridge the primary and secondary care gaps that have caused fragmentation in the Australian health care system. These gaps have been attributed to the complex interplay of health funding and division of responsibilities between the federal, state and local governments for both private and public health services [43]. Risk stratification is based on the knowledge that approximately 3 per cent of the catchment population are complex patients. A further subset of this group is expected to be ‘end-of-life’ patients, and approximately 11 per cent will be ‘diagnosed but stable’ patients who will be encouraged to self-manage their care in consultation with their GP [44].

Programme funding was provided by Queensland Health, the Gold Coast HHS Board and the Gold Coast Primary Health Network for an initial three year proof of concept, which is being carefully evaluated from both economic and process perspectives. If found to be cost-effective, acceptable to patients and professionals and as good as or better than usual care in terms of outcomes, the strategic intent is to scale the programme across the Gold Coast city and ultimately, to other regions. The programme functions as a collaborative partnership between patients, staff specialists (hospitalists), and the network of participating GPs working with local health and community service organisations. All local general practices were invited by letter to participate in the programme, and of approximately 164 known practices, 14 expressed a willingness to participate in the proof of concept. The active population (as defined by the Royal Australian General Practitioners’ criterion of three or more visits to the practice within the last 2 years) across this network is approximately 140,000 people, or 25% of the Gold Coast population. Practice demographics vary, given that participating practices were self-selected, and range from single GP practices to those with ~ 20 GPs. Their patient populations range between 2,000–30,000 patients, and vary according to patient demographics such as age, ethnicity and socio-economic status.

The centrepiece of the model is a Coordination Centre, a designated clinic off site from the hospital and other health services but operated as a legal entity of Gold Coast Hospital and Health Services. The Centre is the hub of the system, providing rapid access to a multidisciplinary primary and specialist health care team for holistic assessments, and, where required, referral to home care and specialist health and social services or GP services. The Coordination Centre also manages the enhanced Information and Communication Technology systems that house clinical informatics, patient registers, referral networks and ultimately, telehealth and remote monitoring capability. Non clinical ‘Service Navigators’ are also integral to the activities of the Coordination Centre, and they provide liaison between patients, families, health care providers, and community services, such as arranging equipment for home monitoring where necessary. Other features of the model include patient-centred and shared decision-making between clinicians, patients and family members, and direct admission to the hospital Medical Admission Unit or inpatient wards for those requiring admission. The Coordination Centre is funded as part of the programme and as such, is a public facility with no costs incurred or billed to the patient. Patients can choose whether to access public or private hospitals or other specialist services with variable costs depending on their level of private insurance coverage. These insurance arrangements are beyond the purview of the programme.

The approach to risk stratification has three key components. First, hospital data going back three years is analysed to identify existing high users. Second, General Practice clinical information is analysed to identify patients with multiple diagnoses, high use of pharmacy or high use of the GPs clinic. Finally, this information is combined and discussed on a multi-professional basis during which the subjective knowledge of GPs is also factored in to identify patients at risk of high utilisation or poor outcomes that may be improved through better coordination. A major investment in time and technical capability has been made to enable the automated matching of hospital and general practice patient records to facilitate this process. Those considered amenable and suitable for the integrated care model are invited to undergo a holistic assessment as the basis for their Shared Care Plan. The holistic assessment process involves four stages. The first, *Evaluation*, determines their capacity to benefit from improved coordination. Under the direction of the Medical Director of the Coordination Centre a diagnostic review is conducted, which can include a medication review, mental health and frailty assessment, establishment of health goals and the need for a Non-Government Organisation or entitlement review. The second step is *Discovery* where the patient meets with the appropriate members of the multidisciplinary team to help tailor their Shared Care Plan to their individual needs. Step three, the *Patient-Centred Care Planning* stage, is a review by the coordination team in collaboration with their GP. The fourth stage is that of *Communication*, where the care coordinator ensures that the patient and family understand and agree with the Shared Care Plan and that all elements of the plan are documented for the Shared Care Record (SCR), the GP, the HHS record, and any other organisations or resources as appropriate, including addressing any guardianship issues such as power of attorney when required. For the initial data linkage between Hospital services and general practices the use of patient-level and identifiable data was approved by HHS executives, which has the legal authority for approval under the creation of Hospital and Health Services (2011) Act [30]. Those patients identified as appropriate for the programme were invited to provide written informed consent to share their health care information among their health care providers, as well as for programme evaluation purposes. The protocol for the evaluation has been approved by GCHHS Human Research Ethics Committee (HREC) and Griffith University HREC (HREC/15/QGC/22; MED/22/15/HREC).

**Figure 1:**
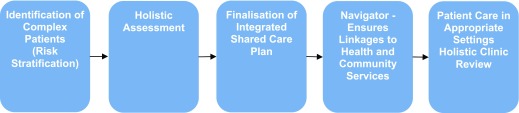
Protocol One: Patients identified as complex and comorbid.

Five protocols guide the process of proactively managing the population. Protocol One (Figure 1), for the high risk patients, begins with a holistic assessment. Next, a set of plans that includes their treatment plan, self-management plan, exacerbation plan, discharge plan and community care support plan are developed. In this protocol, the patient’s lead physician, nurse and navigator are named, and patients are invited to be reviewed at the Coordination Centre or general practice as required, including daily or weekly telephone support or home visits. For those requiring end-of-life care, plans are developed for their pre-terminal care, end-of-life plan, any guardianship issues and bereavement care.

**Figure 2:**
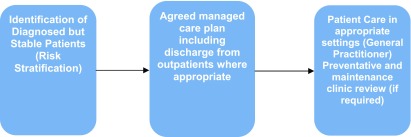
Protocol Two: Diagnosed but stable.

Under Protocol two (Figure 2), patients with stable conditions will continue to have their care managed by their GP as lead physician, supported by clearly articulated pathways, protocols and guidelines. These will identify triggers for situations where a person’s condition deteriorates and specialist intervention is required. In some circumstances they will be attended by allied health practitioners attached to the practice. For those practices without allied health services, or where patients require urgent allied health care unavailable in the practice, they are encouraged to call the Coordination Centre. Where necessary patients will be upgraded to Protocol 1 but generally, this group are supported through the integrated care programme to develop self-management capacity and restorative care through the HHS.

**Figure 3:**
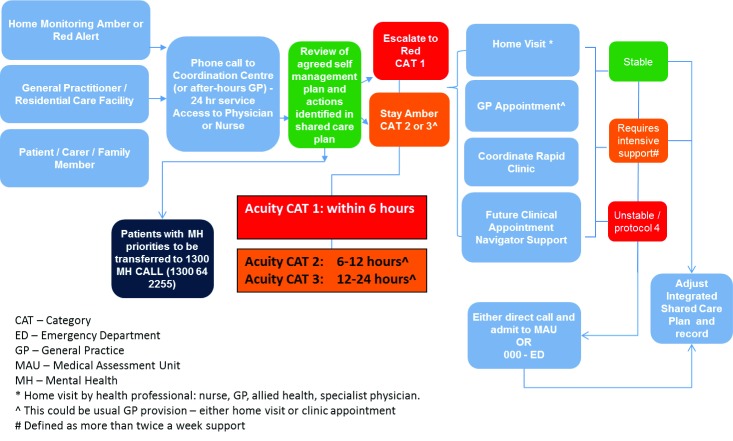
Protocol Three: Exacerbation.

Reactive protocols for unscheduled care have been developed as a rapid response process referred to as Protocol Three (Figure 3). Currently, the Coordination Centre undertakes daily monitoring of admissions and discharges. Patients requiring rapid responses are identified through an electronic alert system as they register at the Emergency Department. The rapid response involves sending a care team from the Coordination Centre to the Emergency Department. As with other patients in the programme, care is supported with daily home or residential care monitoring and ongoing access to review through the Coordination Centre to ensure timely responses with a single point of contact.

**Figure 4:**
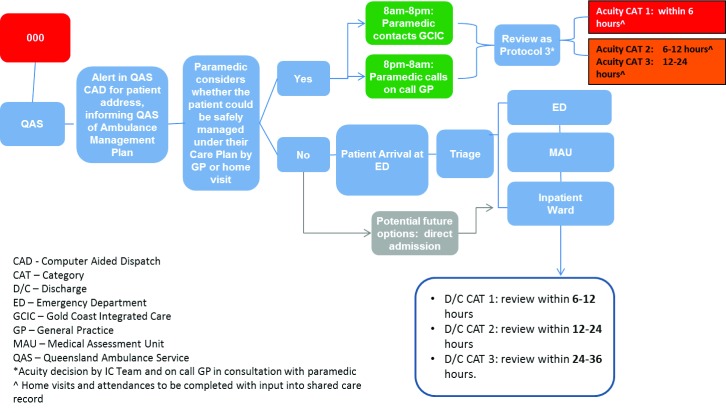
Protocol Four: Emergency.

**Figure 5:**
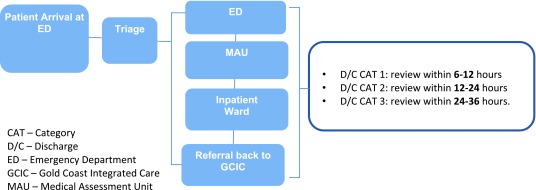
Protocol Five: Self-Presentation to Emergency.

Patients requiring an emergency medical admission to hospital, whether by GP, Coordination Centre or self-referral, may be admitted to the Medical Admission Unit (MAU) directly, bypassing the Emergency Department (see Protocols Four and Five, Figures 4,5). Direct admission will be available for those patients who have had a holistic assessment and care plan established, with an indication that the admission is in relation to an exacerbation of a diagnosed condition. This will be expedited by identifying the urgency of their need through the GP, Coordination Centre, or after hours, through a call centre process.

## Progress with the Gold Coast Integrated Care Model

The programme is now live and beginning the process of identifying the high risk population and ‘on boarding’ patients into the new care system. Fourteen general practices have begun working with the Coordination Centre and the holistic assessment process and systematic register development has commenced, focusing initially on Diabetes, Respiratory, Cardiac and Renal conditions as well as frailty, end of life and residential aged care residents. Evaluation data are being collected on all interactions to capture both staff and patient experiences and perspectives on the new model of care, including satisfaction indicators. Following the recommendations of Petch et al [40] we will be focusing on evaluating changes, including culture change, health outcomes, and a contextualised evaluation of clinical and professional engagement. Our economic evaluation will provide precise costings of the model according to utilisation of various services and cost per patient according to demographic and clinical condition, hospital admissions by Diagnostic Related Group, status on discharge, average bed days per episode per condition, major procedures and the rate of avoidable hospitalisations for those with acute exacerbation of their condition. A control group of matched patients with complex and chronic conditions from the community matched on clinical, demographic and historical hospitalisation patterns, will allow comparisons between the integrated care programme and usual care. These patients are being recruited from lists of patients identified by the HHS as potential matched controls. Approval to access patient level data has been granted by Queensland Health. A random sample of these patients will be invited by mail to consent to and complete the same survey as those already enrolled in the programme. A number of health status indicators will also be analysed in conjunction with GP management, including adherence to clinical guidelines, adverse events, health risks and mortality. Early indicators show enthusiasm for the model by patients, GPs, practice nurses and practice managers.

## Conclusion

The Gold Coast Integrated Care programme is an attempt to synthesise the best available international evidence into a professionally-led design suitable for implementation at scale in the Australian context. We have already overcome a number of obstacles related to the willingness of general practice and sub-specialist teams to participate, organisational appetite for risk and the detailed technical challenges of implementing a shared care record and matching patient records between hitherto unlinked care sectors. As the proof of concept moves across the population, we aim to be offering proactive, shared care for higher risk patients within a population of approximately 140,000 active patients across our General Practice network by April 2016 and will then evaluate the effectiveness of the model for 24 months. We anticipate publishing our full economic and process evaluation following the completion of the pilot programme in 2018.
